# Reliability-Gated Stereo Vision and 2.5D Sensor Fusion for Active Robotic Observation in Occluded Manufacturing Workspaces

**DOI:** 10.3390/s26196106

**Published:** 2026-09-26

**Authors:** Mengjie Li, Le Zhao, Leqi Li, Yuxiao Zhang, Gengpei Zhang

**Affiliations:** 1 School of Electronic Information and Electrical Engineering, Yangtze University, Jingzhou 434023, China; 2024001574@yangtzeu.edu.cn (M.L.);; 2 School of Computer Science, Yangtze University, Jingzhou 434023, China

**Keywords:** stereo vision, reliability gating, sensor fusion, 2.5D mapping, active perception, occluded environment

## Abstract

**Highlights:**

**What are the main findings?**

- Reliability-gated stereo fusion prevents unreliable observations from contaminating the accumulated 2.5D map.
- Compared to the 2D baseline, 2.5D mapping improved the Medium2 success rate from 80% to 100% under the same observation budget.

**What are the implications of the main findings?**

- Weak semantic cues can guide target-oriented observation while preserving geometric feasibility.
- Reliability-aware sensing and height-aware viewpoint planning can improve active robotic observation under occlusion.

**Abstract:**

Active robotic target observation in occluded manufacturing workspaces is difficult when unreliable stereo depth, missing obstacle-height information, and infeasible viewpoint commands propagate through the perception–mapping–planning loop. This study develops a reliability-gated stereo vision and 2.5D fusion framework for active target observation with an eye-in-hand stereo system. The framework screens low-quality stereo observations before fusion, verifies whether accepted observations produce effective map updates, and maintains a 2.5D occupancy-height representation for height-aware viewpoint generation. Using this representation, constrained semantic–geometric ranking selects next-best-view (NBV) candidates, while execution feedback, failed-region avoidance, and anchor recovery maintain closed-loop continuity. Closed-loop simulations are conducted in occluded robotic workcell scenarios with different difficulty levels. The method achieves 100% success in all Easy and Medium scenes. In Medium2, success rates are 80% for the 2D baseline and 100% for the 2.5D representation; the corresponding mean search steps among successful trials are 6.12 ± 0.64 and 3.40 ± 0.70, respectively. Real-scene stereo front-end validation supports depth recovery, quality-gated observation screening, initial 2.5D map fusion, and first-NBV generation under occlusion. These results indicate that reliability-gated stereo fusion and height-aware viewpoint planning can improve active robotic observation in occluded manufacturing workspaces.

## 1. Introduction

Active robotic target observation is a common requirement in automated manufacturing workspaces, robotic inspection, and vision-guided handling. In practical workcells, workpieces, fixtures, tools, and nearby structures can partly or fully occlude the target in the initial camera view. A single passive observation is therefore often insufficient. The system must update the scene representation and choose subsequent viewpoints until the target can be observed reliably. Next-best-view (NBV) planning is therefore central to closed-loop active perception in occluded manufacturing environments [1].

Stereo vision provides spatial information for active observation, but unreliable depth estimates can contaminate the global map and mislead later viewpoint decisions [2,3,4]. This risk is pronounced under weak texture, grazing views, near-field occlusion, and depth discontinuities. A purely 2D map also cannot represent obstacle height, which limits elevated and bypassing viewpoints in occluded scenes. In fixed robotic workcells, candidate viewpoints must satisfy workspace, pose, standoff-distance, and motion-stability constraints. Active target observation therefore couples perception, mapping, planning, and execution.

This study develops a reliability-gated stereo vision and 2.5D fusion framework for active robotic target observation in occluded manufacturing workspaces. The framework rejects low-quality stereo observations before fusion and checks whether accepted observations produce effective global map updates. It maintains a 2.5D occupancy-height map and selects NBV candidates through constrained semantic–geometric ranking. Execution feedback is included through feasibility checking, failed-region avoidance, and anchor recovery, so infeasible motions become explicit constraints in subsequent planning.

The main contributions are as follows.

(1) A reliability-gated observation-to-map fusion strategy is developed for stereo-based active observation under occlusion. Low-quality or ineffective stereo observations are rejected before they contaminate the accumulated 2.5D map.
(2) A height-aware NBV generation strategy is introduced using a 2.5D occupancy-height representation. Compared to a 2D baseline, this representation supports obstacle-top and bypassing candidate generation in occluded manufacturing workspaces.
(3) A constrained semantic–geometric NBV policy with execution feedback is proposed. Weak target cues only re-rank geometrically feasible candidates, while feasibility checking, failed-region avoidance, and anchor recovery support closed-loop continuity.

Closed-loop simulations and real-scene stereo front-end validation are used to evaluate the framework. The simulations assess search success, search efficiency, ablation effects, and the contribution of the 2.5D representation over a 2D baseline. The real-scene validation focuses on depth recovery, quality-gated observation screening, initial 2.5D map fusion, and first-NBV generation under occlusion. Full closed-loop performance is evaluated only in simulation; the real-scene tests do not establish complete robotic deployment performance.

## 2. Related Work

This work builds on active vision and next-best-view planning, reliability-aware map construction under occlusion, and semantically guided target search. The central challenge is to integrate these components into a stable closed-loop active observation framework for constrained manufacturing workspaces.

### 2.1. Active Vision and Next-Best-View Planning

Active vision and next-best-view (NBV) planning improve observation quality by selecting informative viewpoints [5,6,7,8]. Mobile-robot and UAV studies often emphasize frontier expansion, information gain, or 3D reconstruction in large-scale environments [1,9,10,11,12]. These settings differ from fixed robotic workcells, where feasible viewpoints are constrained by reachability, standoff distance, pose range, and motion stability.

For robotic inspection and manipulation, view planning has been studied in active perception and object reconstruction [13,14,15,16]. Related work has also addressed path planning for laser scanning with an industrial robot [17]. Existing methods often use known object models, offline candidate sets, or reachability filtering after viewpoint scoring. Online target observation under unknown occlusion is more demanding because perception quality, target-related cues, geometric visibility, and execution feasibility must be evaluated within the same closed loop [18,19,20].

### 2.2. Reliability-Aware Perception and Map Construction Under Occlusion

Scene representation underpins active viewpoint planning. OctoMap, volumetric fusion, and occupancy-grid methods provide different trade-offs between map completeness and computational cost [21,22,23,24,25,26]. In occluded manufacturing workspaces, dense 3D mapping may be unnecessary for viewpoint selection, while a 2D grid discards obstacle-height information. A 2.5D occupancy-height representation therefore offers a practical compromise because it preserves height cues for obstacle-top and bypassing viewpoint generation while remaining compact for online planning [27,28].

Reliability is equally important. Many stereo-vision studies focus on depth accuracy, disparity estimation, or pixel-level confidence [29,30,31,32,33]. In an active observation loop, reliability must also be assessed at the observation level. The question is not only whether local depth estimates are reliable, but whether the current observation should be fused into the global map. Low-quality stereo observations may introduce pseudo-obstacles or incorrect free-space regions, thereby misleading subsequent NBV decisions [34]. This motivates observation-level screening and effective map-update validation before global fusion.

### 2.3. Semantically Guided Exploration and Target Search

Semantically guided exploration can make viewpoint selection more target-oriented than purely geometry-driven exploration [5,35,36,37,38,39,40,41,42,43]. In occluded manufacturing workspaces, target-related detections are useful but often incomplete, weak, or view-dependent. Semantic cues are therefore better used as directional preferences, not as substitutes for geometric feasibility or perception reliability.

These observations motivate the reliability-gated active observation framework developed in this paper. The framework combines stereo observation screening, effective 2.5D map updating, height-aware candidate generation, weak semantic re-ranking, and execution-feedback constraints. This integration is designed to improve closed-loop target observation under occlusion without relying solely on stereo depth, semantic detection, or nominal viewpoint scores.

## 3. Materials and Methods

Active robotic target observation is formulated as a closed-loop process that couples stereo perception, map updating, viewpoint planning, and execution feedback. The proposed framework is designed to prevent unreliable stereo observations from contaminating the global map, preserve height information for next-best-view generation, and avoid repeated infeasible viewpoint commands during closed-loop observation.

As shown in Figure 1, each observation cycle consists of four main stages. First, the eye-in-hand stereo sensor acquires image pairs, from which dense depth and weak target cues are obtained. Second, the observation is screened before fusion, and only effective updates are integrated into the global 2.5D map. Third, height-aware viewpoint candidates are generated and ranked using semantic–geometric next-best-view planning. Finally, the selected viewpoint is checked and executed. Failed or unstable viewpoints are then fed back to the planner through failed-region blacklisting and anchor recovery. The following subsections describe the problem formulation, reliability-gated map fusion, height-aware viewpoint generation, semantic–geometric NBV selection, and execution-feedback mechanism.

### 3.1. Overview and Problem Formulation

We consider active target observation in an occluded fixed robotic workcell equipped with an eye-in-hand stereo sensor. The target may be partially or fully occluded and may be absent from the initial field of view. Without a global prior, the system alternates between stereo observation, map updating, viewpoint planning, and execution feedback until the target is observed with sufficient confidence.

At observation cycle t, the system receives the current stereo observation $I_{t}$, extracts a local geometric representation $L_{t}$, and maintains a global 2.5D map $M_{t}$. Based on $I_{t}$, $L_{t}$, and $M_{t}$, a constrained candidate set is generated and the next observation pose $V_{t+1}$ is selected. The process terminates when the target-observation confidence reaches the stopping condition. Before formal next-best-view planning, several predefined safe initialization observations are used to establish an initial geometric prior and reduce dependence on a single starting view.

The fixed workcell setting imposes constraints on every stage of the loop. During map updating, only observations within the valid working range are allowed to update the global map. Abnormal near-field or out-of-range measurements are prevented from affecting later planning. During candidate generation, each viewpoint must satisfy workspace, viewing-direction, standoff-distance, candidate-radius, and failed-region constraints. During execution, the selected viewpoint must pass feasibility and arrival checks. Failed or unstable viewpoints are recorded and avoided in subsequent cycles, so execution failures become explicit constraints for later viewpoint selection.

### 3.2. Reliability-Gated Observation-to-Map Fusion

The global map carries scene information across observation cycles, so unreliable stereo observations should not be fused directly into the accumulated representation. Observation-to-map fusion is therefore controlled by two sequential checks: local observation-quality screening and global map-update validation. The first check determines whether the current stereo observation is reliable enough for fusion. The second verifies whether the accepted observation contributes effective information to the global 2.5D map.

For each stereo observation, dense depth is first estimated from the image pair, and local geometry is extracted in the camera frame [30]. Support-plane fitting and relative height estimation are then used to obtain free regions, obstacle regions, and the local height distribution [44]. These local geometric cues provide the basis for quality screening and 2.5D map fusion.

Near-range views, weak texture, occlusion boundaries, and grazing angles may produce disparity tearing, flying points, and isolated pseudo-obstacles [29,34]. The proposed screening step is not intended to improve the stereo estimator itself. Instead, it is used to prevent unreliable observations from contaminating the global map and misleading subsequent next-best-view planning.

Four quality indicators are extracted from local grid processing: valid-depth coverage, support-region continuity, local noise level, and abnormal obstacle height. Let $N_{valid}$ denote the number of valid depth pixels in the current frame, and let $N_{roi}$ denote the number of pixels in the evaluation region. The valid-depth ratio is defined as

(1) $$r_{\mathrm{valid}}=\frac{N_{\mathrm{valid}}}{N_{\mathrm{roi}}}.$$

Let $A_{table}$ denote the total projected area of the support-plane candidate region, and let $A_{table}^{max}$ denote the area of its largest connected component. The support-region continuity ratio is

(2) $$r_{\mathrm{table}}=\frac{A_{\mathrm{table}}^{max}}{A_{\mathrm{table}}}.$$

If no valid support-plane candidate region is extracted, that is, $A_{table}=0$, then $r_{table}$ is set to 0 and the frame is treated as invalid.

Let $A_{obs}$ denote the total area of the local obstacle region, and let $A_{small}$ denote the total area of small connected components below a predefined threshold. The small-component noise ratio is

(3) $$r_{\mathrm{noise}}=\frac{A_{\mathrm{small}}}{A_{\mathrm{obs}}}.$$

The maximum obstacle height relative to the support plane is

(4) $$h_{max}={max}_{p\in P_{\mathrm{obs}}}h\left(p\right),$$

where $P_{obs}$ is the set of points classified as obstacles, and $h\left(p\right)$ is the height of point p relative to the fitted plane. If $P_{obs}$ is empty, $h_{max}$ is set to 0. If $A_{obs}=0$, $r_{noise}$ is also set to 0 to avoid division by zero.

Let $Q_{t}\in \left\{0,\;1\right\}$ denote the local observation-quality flag. The current observation is accepted for global fusion only when

(5) $$Q_{t}=1\;\;⟺\;\;r_{\mathrm{valid}}\geq \tau_{\mathrm{valid}},\;\;r_{\mathrm{table}}\geq \tau_{\mathrm{table}},\;\;r_{\mathrm{noise}}\leq \tau_{\mathrm{noise}},\;\;h_{max}\leq \tau_{h}.$$

Two auxiliary visibility criteria are also used to suppress near-field occlusion: the central-region valid-depth ratio and the obstacle-dominance ratio. If $Q_{t}=0$, the current observation is rejected and the global map remains unchanged. The thresholds and auxiliary visibility criteria used for quality-gated observation screening are summarized in Supplementary Tables S1 and S2. These thresholds were set empirically during preliminary system development to screen insufficient depth coverage, unreliable support-plane estimates, and fragmented or occlusion-dominated observations. They were fixed before the formal experiments and were not retuned for individual test scenes.

Accepted observations are transformed into the planning frame and fused into a global 2.5D occupancy-height map centered at the robotic workcell base [21,27,28]. The map contains two coupled layers: an occupancy layer that records free, occupied, and unknown cells, and a height layer that stores the maximum observed obstacle height in each occupied cell. During fusion, free space is cleared first, obstacle cells are updated according to the maximum observed height, and unobserved cells remain unknown.

A locally reliable observation may still fail to produce a useful global update. For example, valid points may fall outside the effective working range or overlap with already observed regions, leaving the global map almost unchanged. Therefore, a map-update validation flag $U_{t}\in \{0,\;1\}$ is introduced. The flag $U_{t}$ is set to 1 if the accepted observation produces an effective global map update; otherwise, it is set to 0.

The effective-perception flag is then defined as

(6) Pt=Qt ∧Ut.

Here, $Q_{t}$ indicates local observation quality, $U_{t}$ indicates effective global map updating, and $P_{t}$ indicates that the current observation is both locally reliable and globally useful. This flag is used to compute the average effective perception steps and the map-update rate in the experiments.

### 3.3. Height-Aware Candidate Generation

Based on the 2.5D global map, the planner generates candidate observation poses for subsequent next-best-view selection. Frontier points are used as exploration seeds rather than direct target poses [9]. Unknown-region boundaries are extracted from the current global map, and local unknown centers are estimated to guide candidate sampling. Around these regions, standoff candidate poses are generated in forward, backward, lateral, and obstacle-bypassing directions [10,11].

To handle different occlusion structures, three candidate types are considered: free-space views, obstacle-ring views, and obstacle-top views. Free-space views are sampled from valid free regions and provide safe exploratory observations. Obstacle-ring views are generated around obstacle boundaries or unknown-region frontiers to improve lateral visibility. Obstacle-top views use the height layer of the 2.5D map to propose elevated observations above occluding objects, which cannot be obtained from a purely 2D occupancy map [18,28].

The candidate set at observation cycle t is denoted as

(7) $$C_{t}=C_{free}\;\cup C_{ring}\;\cup C_{top},$$

where $C_{free}$, $C_{ring}$, and $C_{top}$ represent free-space, obstacle-ring, and obstacle-top candidates, respectively. Before ranking, candidates that violate workspace, viewing-direction, standoff-distance, height, radius, or failed-region constraints are removed. This filtering prevents clearly infeasible viewpoints from entering the semantic–geometric scoring stage.

Obstacle-height information is essential in occluded manufacturing workspaces. A purely 2D map can support lateral bypassing views, but it cannot distinguish low obstacles from tall occluders or generate elevated viewpoints based on obstacle height. In contrast, the proposed 2.5D representation enables height-aware candidate generation while avoiding the computational cost of dense 3D reconstruction.

### 3.4. Semantic–Geometric NBV Selection

After candidate generation, the planner selects the next observation pose through constrained semantic–geometric ranking. The objective is to choose a viewpoint that can improve target observation while satisfying the geometric and execution-related constraints of the fixed robotic workcell.

For each candidate viewpoint $v\in C_{t}$, a geometric score is computed by combining normalized gains and penalties:

(8) $$S_{geo}\left(v\right)=\sum_{i\in G+}w_{i}^{+}\;g_{i}\;\left(v\right)-\sum_{j\in G-}w_{j}^{-}p_{j}\left(v\right).$$

The positive terms reward unknown-region coverage, exploration need, visibility, over-obstacle observation potential, safe standoff distance, and candidate type. The penalty terms suppress overlap, repeated views, failed views, oscillation, blind zones, and steep pitch. The principal scoring weights are listed in Supplementary Table S3. The scoring weights, including the semantic coefficient, were selected empirically during preliminary development to balance exploration utility against repeated views and motion costs. The same weights were then used across the formal test scenes without scene-specific retuning.

Purely geometric exploration may still miss target-related directions when the target appears only as a weak cue. Semantic information is therefore used only to re-rank geometrically feasible candidates, rather than replacing geometric exploration [5,35,36]. At each perception step, the best target detection from the stereo images is retained. The YOLOv8 detector (Ultralytics, Frederick, MD, USA) is used only as a weak task cue and stopping signal, not as a methodological contribution of this paper. The semantic triggering and stopping thresholds are listed in Supplementary Table S4.

For an image width W and a horizontal field of view $\phi_{h}$, the horizontal angular deviation in the detected target is approximated as

(9) $$\delta_{\mathrm{sem}}=\arctan\left(\frac{2(u^{*}-W/2)}{W}tan(\phi_{h}/2)\right),$$

where $u^{*}$ denotes the horizontal center of the best target detection. Under the zero-roll assumption, the semantic preference direction is

(10) $$\theta_{\mathrm{sem}}=\theta_{\mathrm{cur}}+\delta_{\mathrm{sem}}.$$

The directional consistency between candidate viewpoint v and the semantic preference direction is defined as

(11) $$a\left(v\right)=\cos\left(\theta \left(v\right)-\theta_{\mathrm{sem}}\right).$$

The semantic reward is then defined as

(12) $$S_{\mathrm{sem}}\left(v\right)=\lambda_{\mathrm{sem}}\;c^{*}\;a\left(v\right),$$

where $c^{*}$ is the confidence of the best target detection and $\lambda_{sem}$ is the semantic weighting coefficient. In this study, $\lambda_{sem}=1.35$. The final NBV score is

(13) $$S\left(v\right)=S_{\mathrm{geo}}\left(v\right)+S_{\mathrm{sem}}\left(v\right).$$

In the absence of a retained cue, when $c^{*}<\tau_{trig}$, the semantic term is not activated and $S\left(v\right)=S_{geo}\left(v\right)$. When $c^{*}\geq \tau_{stop}$, the system terminates the active observation loop. Thus, the semantic term provides only a weak directional bias among feasible candidates, while viewpoint selection remains constrained by geometric utility and execution feasibility. A missed or low-confidence detection does not trigger stopping or supply a new semantic cue; a previously retained cue may still guide ranking. When neither a current nor a retained cue is available, planning uses geometric scoring alone. An incorrect cue can bias ranking but does not bypass geometric feasibility checks. These checks do not verify target identity: a high-confidence false positive meeting the stopping threshold can terminate the search prematurely. Temporal or multi-view target confirmation is therefore a direction for future work.

Candidate viewpoints are checked in descending order of $S\left(v\right)$. Candidates that violate workspace limits, viewing-angle constraints, standoff-distance limits, height limits, or failed-region restrictions are discarded. The first feasible candidate is selected as the next NBV command and passed to the execution-feedback module.

### 3.5. Execution Feedback and Closed-Loop Recovery

High-scoring viewpoints may still be difficult to execute in a fixed robotic workcell. The execution layer is therefore incorporated into closed-loop planning rather than treating the selected NBV as a direct controller command. Its role is to preserve observation continuity by checking viewpoint executability, detecting unstable motion, and feeding execution failures back to the planner.

After an NBV command is selected, a staged motion strategy is used to avoid large abrupt pose changes [13,14]. The end effector is first lifted to a safe transition height, then translated and adjusted at that height, and finally aligned and descended near the target observation pose. This staged execution reduces the risk of unstable motion in constrained workcell configurations.

After execution, an arrival check is performed before the system proceeds to the next perception–mapping–planning cycle. Let Δp denote the position error, Δyaw, Δpitch, and Δroll denote the attitude errors, and $n_{stable}$ denote the consecutive stable count. The arrival flag $A_{t}$ is defined as

(14) $$At=1\;\Leftrightarrow \;\left\|\Delta p\right\|\leq \;\tau p,\;\left|\Delta yaw\right|\leq \;\tau yaw,\;\left|\Delta pitch\right|\leq \;\tau pitch,\;\left|\Delta roll\right|\leq \;\tau roll,\;nstable\;\geq \;nmin.$$

In the experiments, the thresholds are set to $\tau_{p}=0.005\;m$, $\tau_{yaw}=1.0{}^{\circ}$, $\tau_{pitch}=1.0{}^{\circ}$, $\tau_{roll}=1.5{}^{\circ}$, and $n_{min}=5$. These thresholds are summarized in Supplementary Table S5. The new observation is accepted as the next valid perception cycle only when $A_{t}=1$.

When inverse-kinematics instability, pose drift, execution timeout, or arrival-check failure occurs, the corresponding viewpoint neighborhood is added to the failure blacklist. The planner then avoids similar high-risk viewpoints in subsequent cycles. If repeated failures occur or no feasible candidate remains, the robot returns to a predefined safe anchor pose and replans from the current global map. The accumulated map is preserved during recovery, allowing active observation to resume from a stable state rather than restart from the initial view.

In this way, execution failures are converted into explicit planning constraints. The execution layer therefore acts not only as a motion interface but also as a feedback mechanism that supports the continuity and stability of the closed-loop active observation process.

## 4. Results and Discussion

The experiments evaluate four aspects of the proposed framework: closed-loop active observation performance, the contribution of individual modules, the effect of the 2.5D representation compared with 2D mapping, and the feasibility of the real-scene stereo front end.

### 4.1. Experimental Setup

Closed-loop simulation experiments were conducted in CoppeliaSim Edu (Coppelia Robotics AG, Zurich, Switzerland) [45] using a UR10 manipulator equipped with an eye-in-hand stereo camera. RAFT-Stereo was used to estimate dense depth from stereo image pairs [30], and a YOLOv8 model with self-trained weights provided weak target cues [46,47]. The detector was kept fixed in all experiments and was used only as a cue source and stopping signal. The simulated stereo camera was constructed by pairing two virtual monocular cameras.

The global scene representation was maintained as a 2.5D occupancy-height map centered at the robotic workcell base. The map size was set to 2.4 m with a resolution of 0.01 m. To reduce unstable stereo fusion in near-field and far-field regions, map fusion was allowed only within a valid working annulus. Regions with radii smaller than 0.35 m were set as free space, regions between 0.35 m and 1.00 m were treated as fusible, and regions outside 1.00 m remained unknown.

The pitch angle was limited to [−60°, −10°], the candidate radius to 0.40–1.00 m, the viewpoint height to 0.05–0.70 m, and the minimum distance to the unknown-region center to 0.18 m. The failed-region blacklist radius was set to 0.04 m. Complete parameter settings are listed in Supplementary Table S6.

Three scene families with increasing occlusion difficulty were evaluated. Easy scenes expose part of the target and usually require only one or two additional observations. Medium scenes place the target behind taller obstacles and require elevated or progressively improved viewpoints. Severe Occlusion scenes hide the target from the initial view and require the planner to combine exploration, weak semantic guidance, and recovery.

For each scene, 10 repeated trials were conducted under the same parameter configuration. The maximum NBV budget was set to eight post-initialization observation steps. A trial was considered successful if the target confidence reached the stopping threshold within this budget.

Supplementary Table S8 reports an initialization-stage computational profile using simulator-generated stereo inputs on the specified laptop. The mean front-end processing time was 3909.6 ms per initialization view, excluding viewpoint motion and settling, of which 3422.5 ms was spent in RAFT-Stereo-based depth processing. This average includes the first invocation, during which the model is loaded before inference. The model is subsequently reused within the same process, so the reported initialization average should not be interpreted as steady-state per-view latency. The additional 779.3 ms corresponds to a single first-NBV decision after six-view initialization, rather than a decision at every view. Here, online observation denotes sequential observation, map updating, and viewpoint selection, not high-frequency real-time control. The intended application is static or quasi-static workspaces where observations can be processed between viewpoint motions; real-time closed-loop performance has not been established. Future work will evaluate implementation optimization and higher-performance computing hardware for reducing latency while preserving reconstruction quality.

### 4.2. Evaluation Metrics

The evaluation metrics include success rate, average search steps, average effective perception steps, final confidence, and effective map-update rate. Let $N_{tr}$ denote the total number of repeated trials,$\;N_{s}$ denote the number of successful trials, and $T_{k}$ denote the number of post-initialization NBV steps used in the k-th trial.

Success rate measures the percentage of trials in which the target is successfully observed within the maximum NBV budget:

(15) $$SR=\frac{N_{s}}{N_{tr}}\times 100\%.$$

Average search steps quantify the number of post-initialization NBV steps required to reach the stopping threshold. This metric is computed over successful trials:

(16) $$T_{avg}=\frac{1}{N_{s}}\sum_{k=1}^{N_{s}}T_{k}.$$

Success rate within the fixed observation budget is the primary task-completion metric. Search steps describe efficiency only among successful trials. When success rates differ, these means may represent different successful subsets and do not alone establish overall superiority. A lower conditional mean does not offset a lower success rate. Failed trials remain included in the success-rate denominator.

Average effective perception steps quantify how many perception steps are both locally reliable and globally useful. This metric is computed over successful trials using the effective-perception flag $P_{t}$ defined in Section 3.2:

(17) $$P_{avg}=\frac{1}{N_{s}}\sum_{k=1}^{N_{s}}\sum_{t=1}^{T_{k}}P_{t}^{\left(k\right)}.$$

Final confidence denotes the target-detection confidence at the end of a trial. For successful trials, it reflects the confidence when the stopping threshold is reached. For failed trials, it records the maximum confidence obtained before the eight-step budget is exhausted.

Effective map-update rate measures the fraction of post-initialization NBV perception steps that produce effective global map updates. It is computed using the map-update validation flag $U_{t}$ defined in Section 3.2:

(18) $$R_{U}=\frac{\sum_{k=1}^{N_{tr}}\sum_{t=1}^{T_{k}}U_{t}^{\left(k\right)}}{\sum_{k=1}^{N_{tr}}T_{k}}\times 100\%.$$

Here, SR is the success rate, $T_{avg}$ is the average search steps, $P_{avg}$ is the average effective perception steps, and $R_{U}$ is the effective map-update rate. Unless otherwise specified, search steps, effective perception steps, and final confidence are reported as mean ± standard deviation over successful trials. Unsuccessful trials are included in the success-rate calculation. The effective map-update rate is computed over all executed post-initialization NBV perception steps.

### 4.3. Closed-Loop Simulation Performance

Table 1 summarizes the closed-loop simulation performance under different occlusion scenes. The proposed framework achieved 100% success in all Easy and Medium scenes and in Severe Occlusion1. In the most challenging Severe Occlusion2 scene, where the target was strongly occluded and effective observations were difficult to maintain, the success rate decreased to 70%.

Among successful trials, the average search steps generally increased with occlusion difficulty. This descriptive trend characterizes completed searches and should be interpreted alongside the success rate, particularly in Severe Occlusion2, where some trials failed within the observation budget. The average effective perception steps and effective map-update rate further show whether these additional observations contributed useful information to the global map. Overall, the results indicate that reliability-gated map fusion, height-aware NBV planning, and execution feedback supported stable active target observation in most occluded workcell scenarios.

Figure 2 shows a representative closed-loop observation process. The system first builds an initial 2.5D occupancy-height map, generates candidate viewpoints from the current map, executes the selected NBV command, and updates the map after the new observation. The loop terminates when the target is observed with sufficient confidence.

Medium2 and Medium3 both achieved 100% success, but Medium2 had a higher effective map-update rate and required fewer average search steps. This suggests that continuous effective updates helped the planner converge to useful viewpoints more quickly. Severe Occlusion2 shows the main limitation of the current framework. When visible regions were extremely limited and effective map updates became discontinuous, the planner had less reliable spatial evidence for viewpoint selection. In this scene, 70% of trials succeeded within the eight-step NBV budget; the reported mean search steps characterize only these successful trials.

### 4.4. Ablation Study

The ablation study evaluates the contribution of quality-gated observation screening, weak semantic guidance, and execution feedback. Each variant removes one component while keeping the other settings unchanged. Table 2 reports the results in Medium2 and Severe Occlusion1, where occlusion makes the differences between variants more evident.

The No quality gate variant in Table 2 disables observation-quality screening within the otherwise unchanged framework, isolating its contribution to the closed-loop evaluation. Removing the quality gate did not reduce the success rate in Medium2 or Severe Occlusion1, but it increased the average search steps. In these tested scenes, the observed benefit is therefore improved search efficiency at the same success rate, rather than an increase in success rate. This result is consistent with the intended role of screening unreliable observations before map fusion. The real-scene rejection examples and fusion comparisons in Supplementary Tables S12 and S13 and Supplementary Figure S5 provide complementary illustrations of this screening mechanism.

The variant without semantic guidance had lower success rates in both evaluated scenes. In Medium2, the success rate decreased from 100% to 80%, and in Severe Occlusion1 it decreased from 100% to 70%. This result indicates that weak target cues help bias geometrically feasible candidates toward target-related directions, especially when the target is only partially visible or appears as a weak detection. However, the semantic term is used only for re-ranking and does not replace geometric exploration.

The variant without execution feedback showed the strongest degradation. In Severe Occlusion1, the success rate decreased from 100% to 50%, and the effective map-update rate decreased from 54.6% to 9.0%. These results indicate that feasibility checking, failure blacklisting, and anchor recovery are important for maintaining closed-loop continuity. Without these mechanisms, the planner may repeatedly select nearby high-risk viewpoints after execution failures, resulting in fewer effective map updates and lower search success.

Initialization was also evaluated, but it is not included in the main ablation table because it mainly affects the early geometric prior rather than the core closed-loop decision mechanism. The initialization results are reported in Supplementary Table S7, and qualitative examples of quality-gated screening, semantic guidance, and initialization are provided in Supplementary Figures S1–S3.

### 4.5. Comparison Between 2D and 2.5D Map Representations

This section evaluates how the 2.5D representation contributes to active target observation under occlusion. The 2D and 2.5D schemes use the same perception pipeline and semantic–geometric scoring framework. Their key difference is that the 2.5D scheme provides explicit obstacle-height information for height-aware candidate generation [21,28].

Figure 3 compares representative NBV planning results in the Medium2 scene. The 2D baseline selected a lateral peeking view based only on planar occupancy. In contrast, the 2.5D planner generated obstacle-top candidates and adjusted the observation height according to the occlusion structure. This comparison shows that the height layer affects both the map representation and the candidate set available for NBV selection.

Table 3 reports quantitative comparisons in Easy2, Medium2, and Severe Occlusion1.

As shown in Table 3, the 2.5D representation achieved a higher observed success rate than the 2D baseline in all three evaluated scenes under the same observation budget. In Medium2, success rates were 80% and 100% for the 2D and 2.5D representations, respectively; mean search steps among successful trials were 6.12 ± 0.64 and 3.40 ± 0.70. In Severe Occlusion1, the corresponding success rates were 50% and 100%, and conditional mean search steps were 6.40 ± 1.14 and 4.80 ± 1.75. The success-rate results support improved task completion in these tested scenes. Because the step statistics describe different successful subsets, they are reported descriptively and are not treated as an unconditional comparison of search efficiency across all trials.

These results indicate that the benefit of the 2.5D representation is not simply due to a higher-dimensional map. Rather, the explicit height layer supplies obstacle-height information for obstacle-top and height-aware viewpoint generation. This advantage became more evident in medium and severe occlusion scenes, where lateral viewpoints generated from a 2D occupancy map may be insufficient for recovering target visibility.

A higher effective map-update rate alone did not necessarily correspond to a higher probability of task completion. For example, the 2D scheme in Easy2 had a higher effective map-update rate than the 2.5D scheme but a lower success rate. Map-update rate should therefore be interpreted alongside task-completion outcomes rather than as a standalone indicator of overall search performance.

### 4.6. Real-Scene Stereo Front-End Validation

This section evaluates whether the proposed stereo front end can process real-scene inputs. The validation focuses on depth recovery, quality-gated observation screening, initial 2.5D map construction, and first-NBV generation. These perception-to-first-viewpoint components are prerequisites for long-horizon online robotic deployment, but they do not constitute a full deployment test.

For real-scene validation, six initialization views were captured with a stereo camera (HBV-1780-2 S2.0; Shenzhen Huiboshijie Technology, Shenzhen, China) in a fixed setup. Depth-derived local grids were fused into an initial global 2.5D occupancy-height map to rank nine predefined first-NBV candidates [30].

Table 4 summarizes four representative real-scene cases. All cases constructed an initial 2.5D map and produced a first NBV. Fewer valid fused views indicate degraded fusion quality under poorer visibility or stronger occlusion.

To quantify real-scene obstacle-height recovery, three paper objects—a large rectangular box (O1), a small rectangular box (O2), and an inverted disposable cup (O3)—were placed at three fixed tabletop positions (P1–P3). Five fresh stereo observations were acquired for each object–position combination while the camera and object remained fixed. Reference heights were measured manually. Each estimate was the median height within a manually selected top-surface ROI, relative to the fitted tabletop plane; the ROI was reused across the five observations in a group. No quality-gate rejection was applied in this offline measurement test. Table 5 reports the resulting errors.

All 45 observations produced valid height estimates. The group-wise MAE ranged from 0.02 to 0.41 cm; the pooled MAE and RMSE were 0.19 and 0.23 cm, respectively. These measurements quantify obstacle-height recovery in manually selected regions under a fixed-camera setup, rather than dense depth-map accuracy or complete fused-map accuracy. The three paper-based objects provide a limited material sample, and this test does not constitute a full robotic closed-loop evaluation.

Figure 4 shows the real-scene stereo front-end validation pipeline, including real-scene capture, stereo input, depth recovery, local map construction, global 2.5D map fusion, and first-NBV generation.

The real-scene examples further show that the first-NBV decision can be generated from geometric evidence alone and re-ranked when weak semantic cues are activated. In the geometry-only case, the semantic bonus is zero and the selected first NBV is determined by exploration gain and safety-related terms. In the semantic-triggered case, the weak target cue changes the ranking order among feasible candidates and selects a more target-oriented first viewpoint. Detailed geometry-only and semantic-triggered ranking examples are provided in Supplementary Tables S10 and S11, and the corresponding real target cue is shown in Supplementary Figure S4.

The quality-gated screening results also show that degraded views can be rejected before global fusion. Even when some initialization views are rejected because of obstacle-dominant observations or low central-region visibility, the system can still construct an initial 2.5D map and generate the first NBV using the remaining valid observations. Detailed rejected views and rejection reasons are reported in Supplementary Table S12. Additional screening results are summarized in Supplementary Table S13, and representative fusion comparisons are provided in Supplementary Figure S5.

Overall, the real-scene results show that the proposed stereo front end can support depth recovery, quality-gated observation screening, initial 2.5D fusion, semantic-guided first-NBV ranking, and degraded-view rejection under real inputs. These results complement the closed-loop simulation experiments by validating the perception and first-viewpoint generation components in real scenes. The current real-scene validation remains limited to the stereo front end and first-NBV generation; long-horizon online robotic deployment remains future work.

## 5. Conclusions

This paper presented a reliability-gated stereo vision and 2.5D fusion framework for active robotic target observation in occluded manufacturing workspaces. The framework couples quality-gated observation screening, map-update validation, 2.5D occupancy-height mapping, semantic–geometric NBV planning, and execution feedback within a closed-loop perception–mapping–planning process. By rejecting unreliable stereo observations, preserving obstacle-height information, and feeding execution failures back to the planner, the method improves the stability of active observation under occlusion.

Closed-loop simulations showed that the framework achieved 100% success in all Easy and Medium scenes and in Severe Occlusion1. Compared with the 2D baseline, the 2.5D representation improved the Medium2 success rate from 80% to 100% under the same observation budget; mean search steps among successful trials were 6.12 ± 0.64 and 3.40 ± 0.70 for the 2D and 2.5D representations, respectively. The real-scene stereo front-end validation showed that the perception-to-first-viewpoint pipeline can support depth recovery, quality-gated observation screening, initial 2.5D fusion, and first-NBV generation under occlusion. The current comparisons are limited to configurations within the proposed framework and do not establish superiority over existing NBV planners. An external quantitative comparison remains necessary, with careful alignment of map representations, action constraints, and task assumptions. Future work will extend this front-end validation toward long-horizon online robotic deployment, including semi-dynamic workspaces with changing occlusions. Comparisons with full 3D volumetric representations under matched task conditions will assess the trade-offs between representational detail, memory use, and computation. Lightweight depth-processing implementations will also be evaluated for latency and reconstruction accuracy.

## Figures and Tables

**Figure 1:**
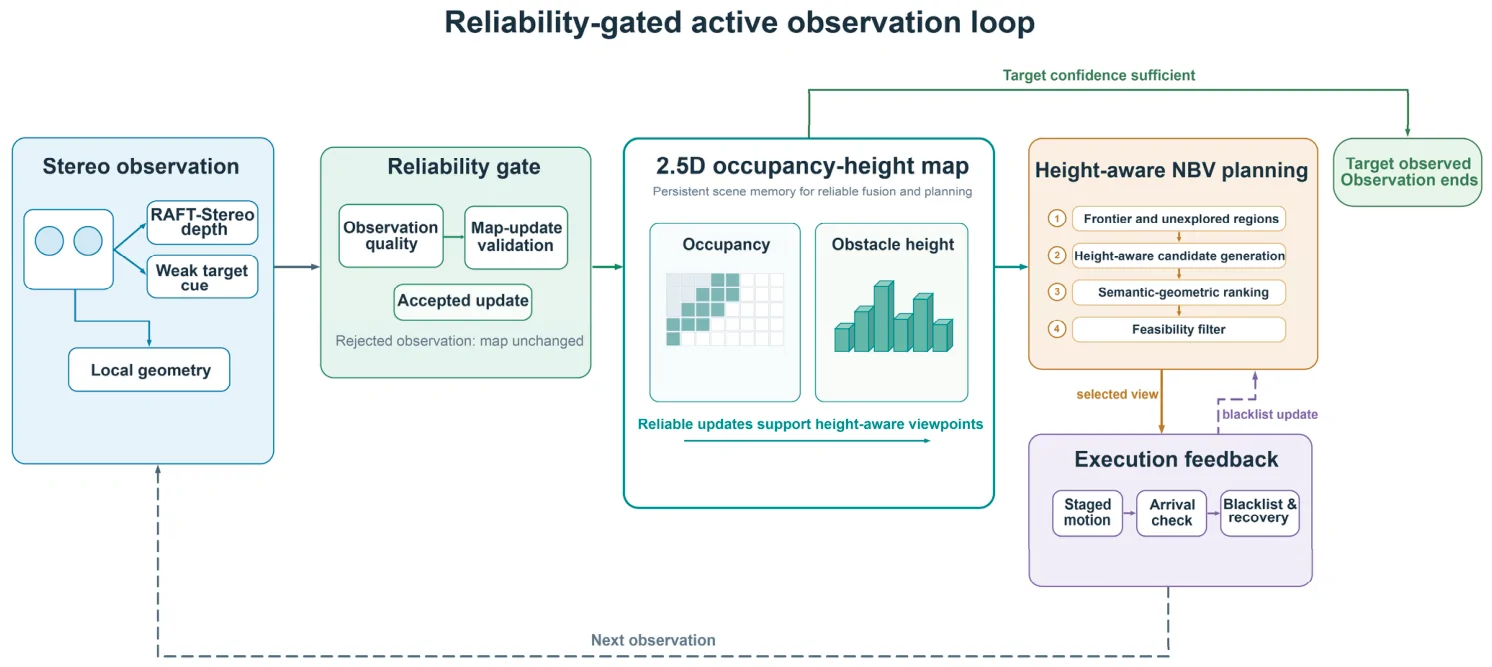
Overview of the proposed reliability-gated active robotic observation framework. The framework integrates stereo perception, reliability-gated 2.5D mapping, NBV planning, and execution feedback into a closed-loop observation process. Low-quality observations are screened before map fusion, and infeasible viewpoints are handled through feasibility checking, failed-region blacklisting, and anchor recovery.

**Figure 2:**
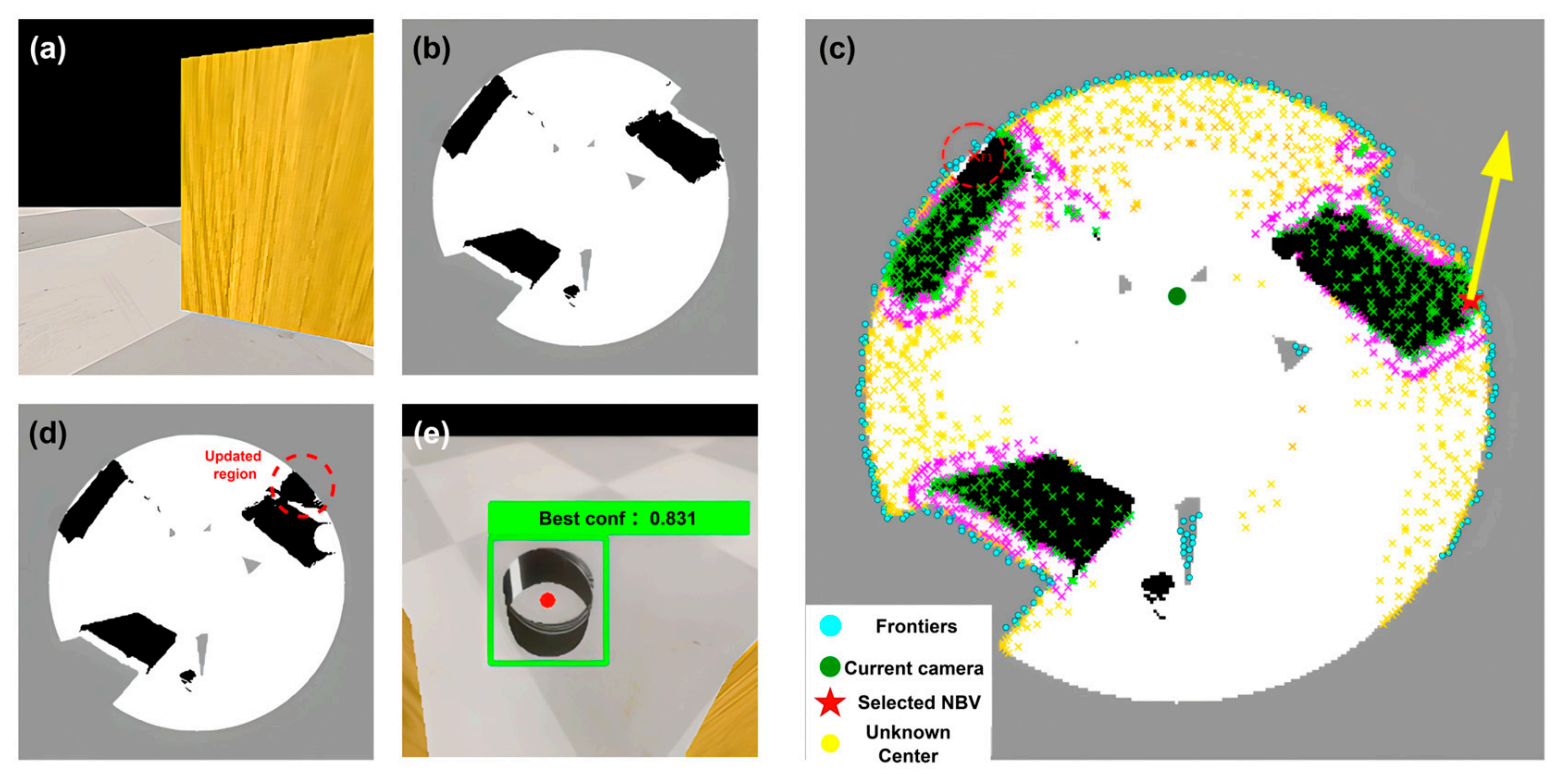
Closed-loop active observation process in a representative occluded scene: (**a**) initial observation; (**b**) 2.5D map after initialization; (**c**) candidate viewpoints and selected NBV; (**d**) map update after executing the selected viewpoint; (**e**) target detection and termination. The red dashed circle in panel (**c**) marks a previously visited observation position at which the target was not detected.

**Figure 3:**
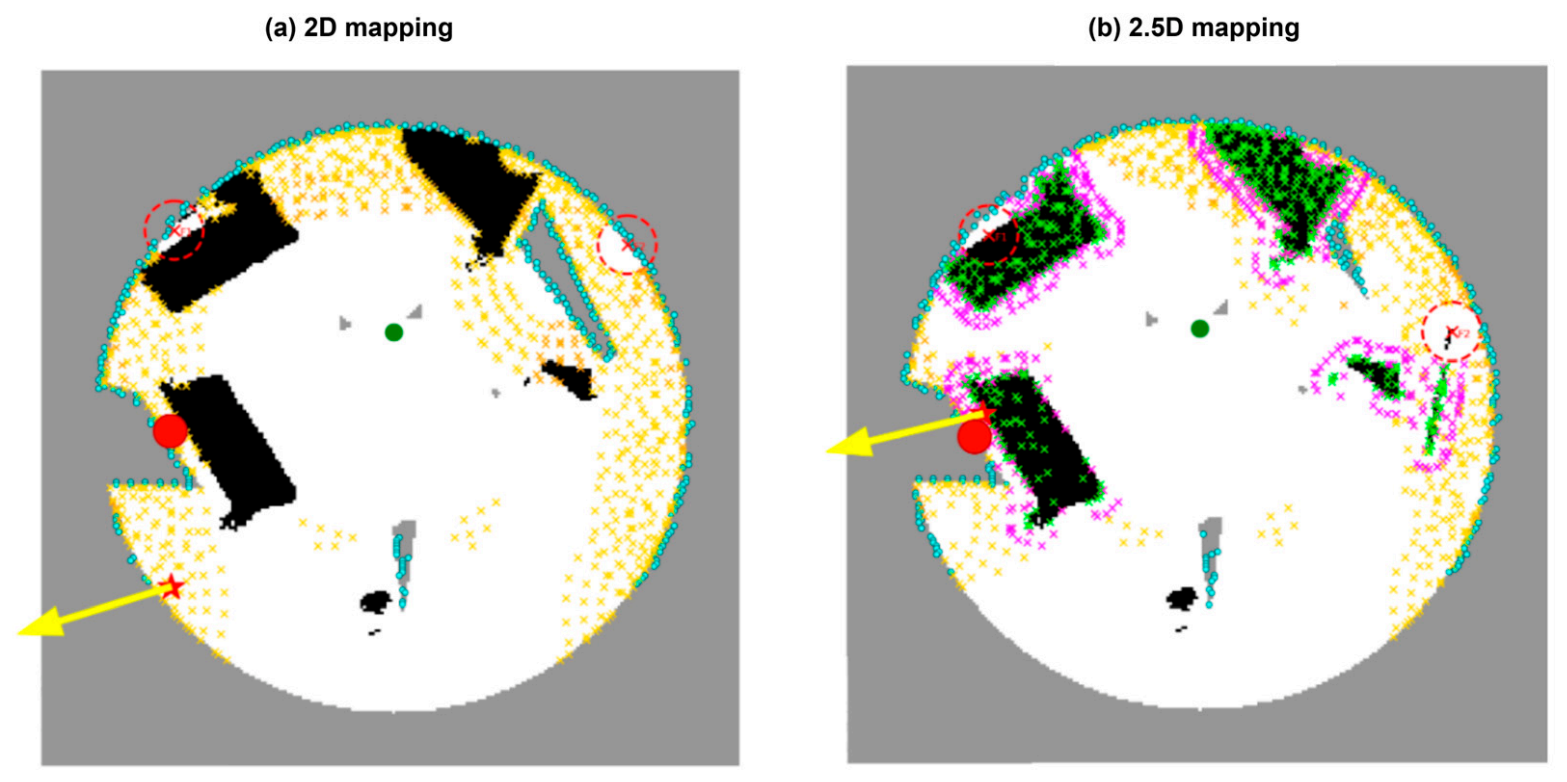
Comparison of NBV planning results under 2D and 2.5D map representations in the Medium2 scene: (**a**) 2D mapping selects a lateral peeking view with a fixed height strategy; (**b**) 2.5D mapping generates an obstacle-top view with adaptive height selection. The red solid circle marks the true target position, the red star marks the selected observation viewpoint, and the yellow arrow indicates the viewing direction.

**Figure 4:**
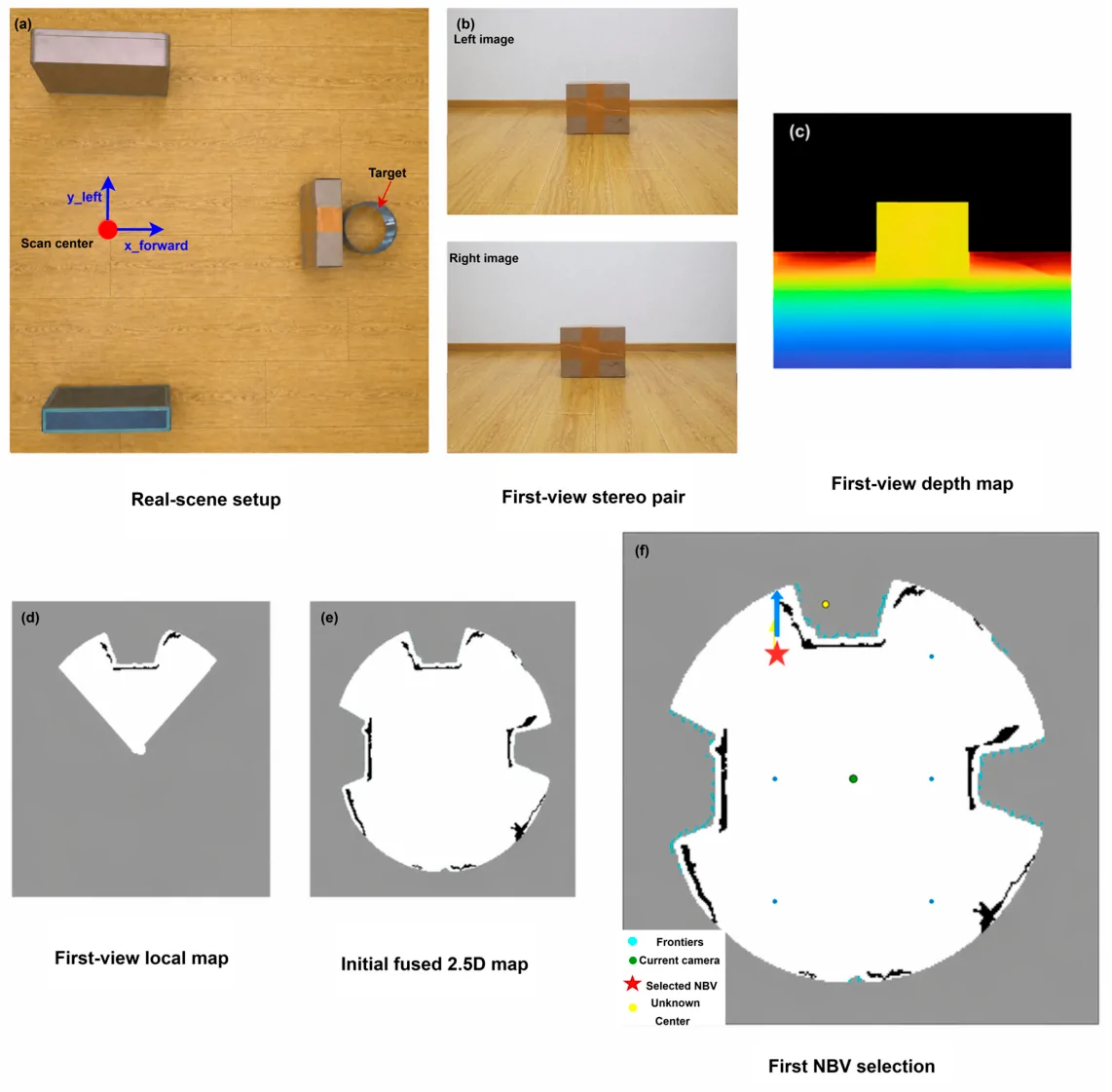
Overview of the real-scene stereo front-end validation: (**a**) real-scene setup; (**b**) first-view stereo pair; (**c**) first-view depth map; (**d**) first-view local map; (**e**) initial fused 2.5D map; (**f**) first NBV selection. The arrow in panel (**f**) indicates the viewing direction selected for the camera’s next observation.

**Table 1:** Closed-loop active observation performance under different occlusion scenes.

Scenario	Success Rate	Avg. Search Steps	Avg. Effective Perception Steps	Effective Map-Update Rate (%)	Final Confidence
Easy1	100%	0.00 ± 0.00	0.00 ± 0.00	—	0.850 ± 0.000
Easy2	100%	1.00 ± 0.00	0.11 ± 0.33	11.1 ± 33.3	0.806 ± 0.025
Medium1	100%	2.00 ± 0.00	1.22 ± 0.36	50.0 ± 0.0	0.786 ± 0.007
Medium2	100%	3.40 ± 0.70	2.20 ± 0.42	65.2 ± 6.4	0.827 ± 0.030
Medium3	100%	3.80 ± 1.03	1.80 ± 1.03	29.1 ± 11.8	0.829 ± 0.004
Severe Occlusion1	100%	4.80 ± 1.75	2.60 ± 0.63	54.6 ± 6.4	0.836 ± 0.022
Severe Occlusion2	70%	6.57 ± 1.40	3.29 ± 0.49	35.8 ± 10.9	0.785 ± 0.017

Note: Success Rate is computed over all repeated trials. Other metrics are reported as mean ± standard deviation over successful trials. A dash indicates that the target is observed before any post-initialization NBV step.

**Table 2:** Ablation results in the Medium2 and Severe Occlusion1 scenes.

Variant	Scene	Success Rate (%)	Avg. Search Steps	Effective Map-Update Rate (%)	Final Confidence
No quality gate	Medium2	100	5.60 ± 1.35	43.3 ± 12.0	0.808 ± 0.025
No quality gate	Severe Occlusion1	100	6.00 ± 0.00	57.3 ± 8.4	0.807 ± 0.010
No semantic guidance	Medium2	80	5.00 ± 1.07	39.8 ± 12.0	0.815 ± 0.012
No semantic guidance	Severe Occlusion1	70	5.43 ± 2.07	41.6 ± 10.1	0.833 ± 0.029
No execution feedback	Medium2	90	4.78 ± 1.30	11.1 ± 4.7	0.815 ± 0.003
No execution feedback	Severe Occlusion1	50	6.80 ± 1.30	9.0 ± 2.3	0.825 ± 0.020
Full model	Medium2	100	3.40 ± 0.70	65.2 ± 6.4	0.827 ± 0.030
Full model	Severe Occlusion1	100	4.80 ± 1.75	54.6 ± 6.4	0.836 ± 0.022

Note: Results are from 10 repeated trials per scene. Metrics other than Success Rate are reported as mean ± standard deviation over successful trials.

**Table 3:** Cross-scene comparison between 2D and 2.5D map representations.

Scenario	Representation	Success Rate (%)	Avg. Search Steps	Effective Map-Update Rate (%)	Final Confidence
Easy2	2D	90	1.67 ± 0.50	40.7 ± 36.4	0.796 ± 0.009
Easy2	2.5D	100	1.00 ± 0.00	11.1 ± 33.3	0.806 ± 0.025
Medium2	2D	80	6.12 ± 0.64	24.3 ± 2.9	0.793 ± 0.029
Medium2	2.5D	100	3.40 ± 0.70	65.2 ± 6.4	0.827 ± 0.030
Severe Occlusion1	2D	50	6.40 ± 1.14	56.4 ± 7.5	0.821 ± 0.026
Severe Occlusion1	2.5D	100	4.80 ± 1.75	54.6 ± 6.4	0.836 ± 0.022

Note: The two schemes share the same perception pipeline and semantic–geometric scoring framework. The 2.5D scheme additionally enables height-aware candidate generation. Metrics other than Success Rate are reported as mean ± standard deviation over successful trials.

**Table 4:** Representative results of real-scene stereo front-end validation.

Scene ID	Scene Condition	Semantic Cue Activated	Valid Fused Views	Selected First NBV (Type)
Scene 1	Clear visibility	No	6	left_front (fixed_free)
Scene 2	Heavy occlusion	No	6	right_40 (obstacle_top)
Scene 3	Severe occlusion	No	4	left_front (obstacle_top)
Scene 4	Limited visibility	Yes	5	left_front (obstacle_top)

Note: All cases in Table 4 used the same six-view initialization scan. “Valid Fused Views” denotes the number of views that passed quality-gated observation screening and were fused into the initial global 2.5D map. Complete results are reported in Supplementary Table S9.

**Table 5:** Quantitative obstacle-height recovery under real-scene stereo input.

Object	Position	Reference Height (cm)	Estimated Height (cm)	MAE (cm)	RMSE (cm)	Available/Attempted
O1	P1	10.00	10.13 ± 0.07	0.13	0.14	5/5
O1	P2	10.00	10.01 ± 0.08	0.06	0.07	5/5
O1	P3	10.00	10.27 ± 0.03	0.27	0.27	5/5
O2	P1	6.50	6.51 ± 0.02	0.02	0.02	5/5
O2	P2	6.50	6.18 ± 0.02	0.32	0.32	5/5
O2	P3	6.50	6.65 ± 0.01	0.15	0.15	5/5
O3	P1	7.60	7.64 ± 0.06	0.05	0.07	5/5
O3	P2	7.60	7.19 ± 0.06	0.41	0.42	5/5
O3	P3	7.60	7.88 ± 0.01	0.28	0.28	5/5
Overall	45 observations	–	–	0.19	0.23	45/45

Note: All three test objects were paper-based. P1–P3 denote fixed placements within the calibrated field of view. Estimated height is reported as mean ± standard deviation over five fixed-setup technical repeats. All selected top-surface regions had 100% valid-depth coverage. Availability denotes a successful plane fit and at least 30 valid ROI pixels, not an error-tolerance test.

## Data Availability

The data supporting the findings of this study are included in the article and its Supplementary Materials. Additional data are available from the corresponding author upon reasonable request. The code used in this study is available from the corresponding author upon reasonable request.

## Supplementary Materials

The following supporting information can be downloaded at https://www.mdpi.com/article/10.3390/s26196106/s1, Table S1: Quality-gate thresholds; Table S2: Auxiliary visibility criteria; Table S3: Semantic-geometric scoring weights; Table S4: Semantic triggering and stopping thresholds; Table S5: Arrival-check thresholds; Table S6: Complete simulation and system parameters; Table S7: Effect of the initialization strategy in Easy2 and Medium2; Figure S1: Effect of quality-gated observation screening on global map quality; Figure S2: Effect of semantic guidance under weak target cues; Figure S3: Effect of initialization strategy on early map priors and observation behavior; Table S8: Hardware details and runtime breakdown; Table S9: Complete real-scene validation results; Figure S4: Target detection result under real stereo observation; Figure S5: Effect of quality-gated observation screening on real-scene map fusion; Table S10: Representative first-NBV candidate ranking without semantic guidance; Table S11: Representative first-NBV candidate ranking with semantic triggering; Table S12: Rejected real-scene views and rejection reasons; Table S13: Quality-gated observation screening results in real scenes.

## Abbreviations

The following abbreviations are used in this manuscript:

NBV	Next-Best-View
YOLO	You Only Look Once
