# Supplementary material for: Reliability-Gated Stereo Vision and 2.5D Sensor Fusion for Active Robotic Observation in Occluded Manufacturing Workspaces
Source: Sensors (Basel). 2026 Sep 26;26(19):6106. doi: 10.3390/s26196106 (PMC13667409; doi:10.3390/s26196106)
Supplement: Supplementary file 1 [file sensors-26-06106-s001.zip › sensors-4544227-supplementary.pdf]

Supplementary Information

Title:

Reliability-Gated Stereo Vision and 2.5D Sensor Fusion for Active Robotic Observation in Occluded Manufacturing Workspaces

Journal:
Sensors

Authors:
Mengjie Li[a], Le Zhao[a], Leqi Li[a], Yuxiao Zhang[b], and Gengpei Zhang[a],*

Affiliations:

[a] School of Electronic Information and Electrical Engineering, Yangtze University, Jingzhou 434023, Hubei, China

[b] School of Computer Science, Yangtze University, Jingzhou 434023, Hubei, China

Corresponding author:
Gengpei Zhang
School of Electronic Information and Electrical Engineering, Yangtze University, Jingzhou 434023, Hubei, China
Email: judgebill@126.com

Supplementary material contents:
This file contains Supplementary Tables S1–S13 and Supplementary Figs. S1–S5 supporting the manuscript.

Supplementary Table S1. Quality-gate thresholds

Category	Metric	Threshold	Description
Plane fitting	Plane fitting status (plane_ok)	True	Frames with failed plane fitting are directly rejected
Depth quality	Valid-depth ratio (valid_depth_ratio)	≥ 0.08	Frames with insufficient valid depth are rejected
Plane quality	Table ratio (table_ratio)	≥ 0.15	Frames with insufficient planar support are rejected
Noise level	Small-component ratio (small_component_ratio)	≤ 0.55	Frames dominated by fragmented obstacle noise are rejected
Height abnormality	Raw maximum obstacle height (max_obstacle_height_raw_m)	≤ 1.5 m	Frames with abnormal obstacle height are rejected
Central visibility	Central ROI valid ratio (roi_valid_ratio)	≥ 0.28	Frames with extremely low central visibility are rejected
Obstacle-dominant view	Combined condition	roi_valid_ratio < 0.50 and obs_ratio_all > 0.28	Frames dominated by close obstacles in the central field of view are rejected

Note: These thresholds are used to identify unreliable observations before global map fusion. A frame is rejected when plane fitting fails, when valid depth or planar support is insufficient, when obstacle fragmentation is excessive, when abnormal obstacle height is detected, or when the central field of view is severely blocked by nearby obstacles.

Supplementary Table S2. Auxiliary visibility criteria

Rule	Condition	Action
Plane fitting failure	plane_ok = False	Reject the frame
Low central visibility	roi_valid_ratio < 0.28	Reject the frame
Obstacle-dominant view	roi_valid_ratio < 0.50 and obs_ratio_all > 0.28	Reject the frame
No valid table region	A_table = 0	Set r_table = 0 and reject the frame
No obstacle region	A_obs = 0	Set r_noise = 0 for the noise-ratio test

Note: These auxiliary rules complement the main quality thresholds in Supplementary Table S1. They reject frames with failed plane fitting, poor central visibility, obstacle-dominant observations, or invalid support-plane regions before global map fusion.

Supplementary Table S3. Semantic-geometric scoring weights

Category	Scoring term	Weight	Description
Positive term	Unknown-region coverage gain	2.10	Encourages candidate viewpoints that cover more unknown regions
Positive term	Exploration need	1.90	Encourages motion toward regions with stronger exploration demand
Positive term	View clarity bonus	1.20	Rewards candidates with a clearer line of sight toward the unknown region
Positive term	Over-obstacle bonus	1.35	Rewards candidates that can observe the scene from above local obstacles
Positive term	Safe standoff bonus	0.60	Encourages candidates that keep an appropriate distance from the unknown-region center
Positive term	Candidate-type bonus	0.80	Rewards candidate types that are more suitable for the current occlusion structure
Penalty term	Coverage-overlap penalty	2.40	Penalizes candidates whose visible regions highly overlap with previously observed areas
Penalty term	Frontier-streak penalty	1.70	Penalizes repeatedly exploring the same frontier region over consecutive steps
Penalty term	View-repeat penalty	1.50	Penalizes candidates with viewing directions similar to recent observations
Penalty term	Failed-view penalty	2.20	Penalizes candidates that are close to previously failed viewpoints
Penalty term	Local-oscillation penalty	1.60	Suppresses repeated oscillation in a small local area
Penalty term	Blind-zone penalty	1.80	Penalizes candidates affected by near-field blind regions
Penalty term	Steep-pitch penalty	0.85	Penalizes candidates with overly steep viewing pitch angles

Note: The semantic term is defined dynamically as

$$S_{sem}(v_i) = \lambda_{sem}\alpha(conf)\cos(\theta_i - \theta_{sem}),$$

where $\lambda_{sem} = 1.35$. Therefore, the semantic bonus is not listed as a fixed scalar weight in the table. It is determined jointly by the semantic weight, the normalized detection confidence, and the directional consistency between the candidate viewpoint and the semantic guidance direction.

Supplementary Table S4. Semantic triggering and stopping thresholds

Parameter	Implementation term	Value
YOLO confidence threshold	YOLO_CONF_THRES	0.25
Semantic cue activation / trigger threshold	semantic_trigger_conf	0.25
Stopping confidence threshold	semantic_stop_conf	0.75
Semantic weighting coefficient	λ_sem	1.35

Note: Semantic guidance is activated when target confidence reaches semantic_trigger_conf and is used only to re-rank geometrically feasible candidates. Search stops when target confidence reaches semantic_stop_conf. Geometric workspace and candidate constraints are reported in Supplementary Table S6.

Supplementary Table S5. Arrival-check thresholds

Item	Value
position tolerance	0.005 m
yaw tolerance	1.0°
pitch tolerance	1.0°
roll tolerance	1.5°
stable count	5

Note: Recover-to-anchor verification uses a looser acceptance setting: 0.015 m, 2.5°, 2.5°, 3.0°, and a stable count of 3.

Supplementary Table S6. Complete simulation and system parameters

Category	Parameter	Value	Description
Global map	Map size	2.4 m	Side length of the global 2.5D map
Global map	Map resolution	0.01 m	Spatial resolution of the global map
Global map	Inner free radius	0.35 m	Regions with radius < 0.35 m are forced as free space
Global map	Fusible radius range	0.35–1.00 m	Regions within this annulus are eligible for map fusion
Global map	Outside region treatment	radius > 1.00 m: unknown	Regions outside the fusion limit remain unknown
Candidate constraints	Pitch range	−60° to −10°	Allowed pitch range of candidate viewpoints
Candidate constraints	Candidate radius range	0.40–1.00 m	Allowed planar distance from the robotic workcell base
Candidate constraints	Viewpoint height range	0.05–0.70 m	Allowed candidate viewpoint height
Candidate constraints	Minimum distance to unknown-region center	0.18 m	Minimum safe standoff distance
Execution constraints	Failed-region blacklist radius	0.04 m	Radius used for failed-region avoidance
Candidate generation	Angular sampling step	12°	Angular interval used for 360° candidate sampling
Candidate generation	Arc-distance samples	0.18, 0.24, 0.30, 0.36, 0.42, 0.50 m	Distance samples around the unknown-region center
Experiment protocol	Repeated trials	10 per scene	Repeated trials under the same parameter configuration
Experiment protocol	Maximum NBV budget	8 post-initialization steps	Maximum number of NBV observations after initialization
Execution constraints	Strict checker	Enabled	Feasibility validation before viewpoint execution
Execution constraints	Failed blacklist	Enabled	Failed candidate regions are added to the blacklist
Execution constraints	Recover-to-anchor	Enabled	Return to a safe anchor after severe execution failure
Reliable perception	Bad-frame rejection	Enabled	Low-quality observations are excluded from fusion
Initialization	Initialization mode	Full	Full initialization scanning strategy
Initialization	Number of initialization viewpoints	9	Viewpoints used in simulation initialization
Initialization	Minimum valid initialization observations	6	Minimum valid observations before entering the NBV stage

Note: Map geometry, outside-region treatment, candidate constraints, repeated trials, and the maximum NBV budget agree with the current manuscript. Semantic triggering and stopping parameters are reported separately in Supplementary Table S4.

Supplementary Table S7. Effect of the initialization strategy in Easy2 and Medium2

Variant	Scene	Success Rate (%)	Avg. Search Steps	Effective Map-Update Rate (%)	Final Confidence
No Initialization	Easy2	70	5.86 ± 1.95	77.3 ± 19.4	0.806 ± 0.026
No Initialization	Medium2	80	5.62 ± 1.85	65.1 ± 24.1	0.791 ± 0.033
Full Model	Easy2	100	1.00 ± 0.00	11.1 ± 33.3	0.806 ± 0.025
Full Model	Medium2	100	3.40 ± 0.70	65.2 ± 6.4	0.827 ± 0.030

Note: Results are reported as mean ± standard deviation over successful trials. The initialization strategy mainly affects the early geometric prior and initial observation behavior, rather than the core closed-loop decision mechanism.

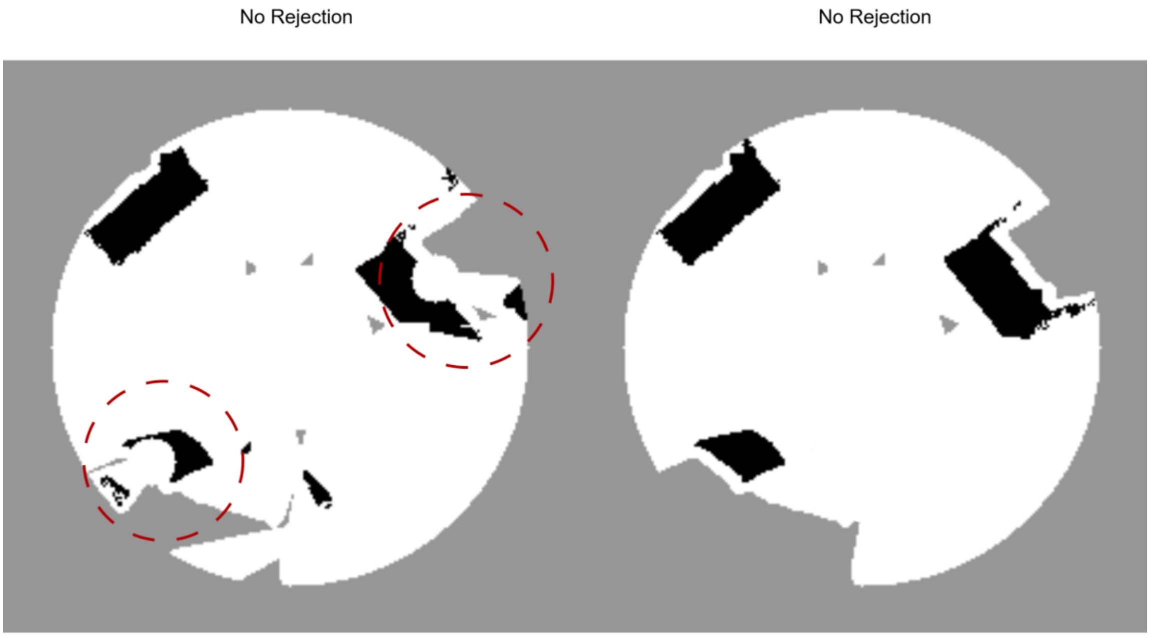

Supplementary Figure S1. Effect of quality-gated observation screening on global map quality.

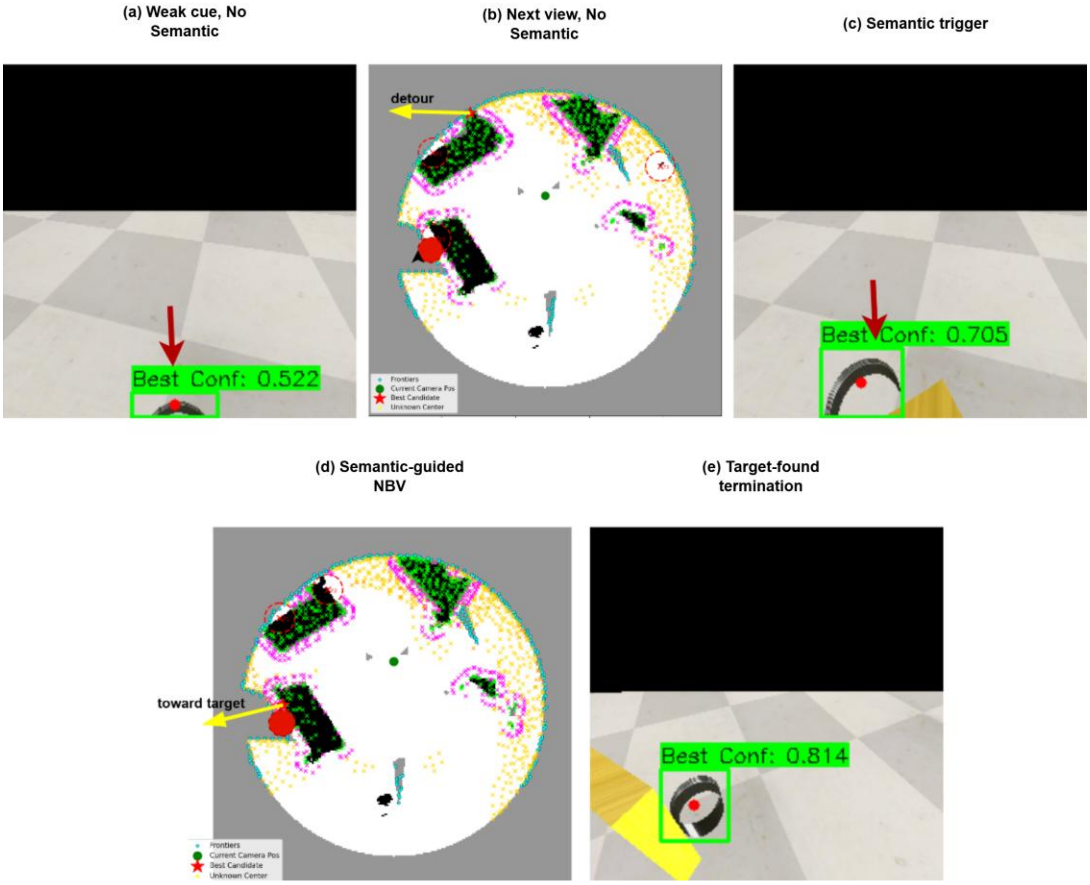

Supplementary Figure S2. Effect of semantic guidance under weak target cues.

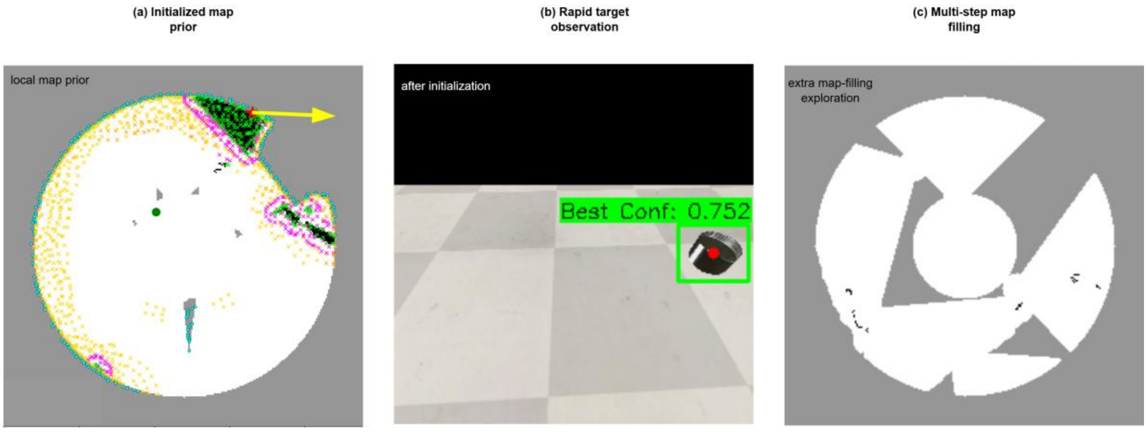

Supplementary Figure S3. Effect of initialization strategy on early map priors and observation behavior.

Supplementary Table S8. Hardware details and runtime breakdown

Section	Item / Processing stage	Detail / Core module	Value / Note
Validation setup	Validation task	Real-scene stereo front-end validation	Confirmed
Validation setup	Computer platform	Lenovo Legion Y7000P laptop	Confirmed
Validation setup	CPU	13th Gen Intel(R) Core(TM) i7-13620H	Confirmed
Validation setup	RAM	16 GB	Confirmed
Validation setup	GPU	NVIDIA GeForce RTX 4060 Laptop GPU, 8 GB VRAM	Confirmed
Validation setup	NVIDIA driver	566.24	Confirmed
Validation setup	Python	3.10.19	Confirmed
Validation setup	PyTorch	1.12.1+cu113	Confirmed
Validation setup	CUDA used by PyTorch	11.3	Confirmed
Validation setup	CUDA acceleration	Enabled	Confirmed
Validation setup	Stereo input	Stereo camera under a fixed observation setup	Confirmed setup
Validation setup	Depth estimator	RAFT-Stereo	Confirmed
Validation setup	Detector	YOLOv8 with self-trained weights	Confirmed
Validation setup	Processing components	Stereo depth recovery; quality-gated observation screening; initial 2.5D fusion; first-NBV decision	Confirmed workflow
Runtime breakdown	Image acquisition	capture_once_and_save	31.041 ms; 0.79%
Runtime breakdown	Configuration update	augment_pipeline_config	1.373 ms; 0.04%
Runtime breakdown	Depth estimation	RAFT-Stereo	3422.544 ms; 87.54%
Runtime breakdown	Local grid construction	RANSAC + grid projection	31.715 ms; 0.81%
Runtime breakdown	Global map fusion	global_map_fusion	161.436 ms; 4.13%
Runtime breakdown	Semantic detection	YOLO	261.439 ms; 6.69%
Runtime breakdown	Complete front-end processing chain	total_without_rig_motion	3909.559 ms; 100.00%
Runtime breakdown	First-NBV decision after six-view initialization	run_nbv_once	779.259 ms; N/A

Note: Runtime values are reported in milliseconds. The complete real-scene front-end processing chain took 3909.6 ms on average and was mainly dominated by RAFT-Stereo depth estimation. The first-NBV decision after six-view initialization took 779.3 ms. The runtime environment used Python 3.10.19 and PyTorch 1.12.1+cu113 with CUDA acceleration on an NVIDIA GeForce RTX 4060 Laptop GPU.

Supplementary Table S9. Complete real-scene validation results

Scene ID	Scene Condition	Semantic Cue Activated	Valid Fused Views	Selected First NBV	First NBV Type
Scene 1	Clear visibility	No	6	left_front	fixed_free
Scene 2	Heavy occlusion	No	6	right_40	obstacle_top
Scene 3	Severe occlusion	No	4	left_front	obstacle_top
Supp-1	Severe occlusion with limited visibility	No	4	front_40	fixed_unknown
Supp-2	Severe occlusion with clearly limited visibility	No	3	right_40	fixed_free
Scene 4	Limited visibility	Yes	5	left_front	obstacle_top
Supp-3	Semantic cue triggered under partially limited visibility	Yes	4	front_40	fixed_unknown
Supp-4	Semantic cue triggered under partially limited visibility	Yes	5	left_front	fixed_unknown
Supp-5	Semantic cue triggered with limited visibility	Yes	4	front_40	fixed_unknown
Supp-6	Semantic cue triggered under severe occlusion and clearly limited visibility	Yes	3	front_40	fixed_unknown

Note: Scene identifiers follow the main manuscript real-scene validation table. Real-A, Real-B, Real-C, and Real-D correspond to Scene 1 (Clear visibility), Scene 2 (Heavy occlusion), Scene 3 (Severe occlusion), and Scene 4 (Limited visibility), respectively. All listed cases built the initial global 2.5D map and generated the first NBV. Valid Fused Views denotes the number of initialization views that passed quality-gated observation screening and were fused into the initial global map.

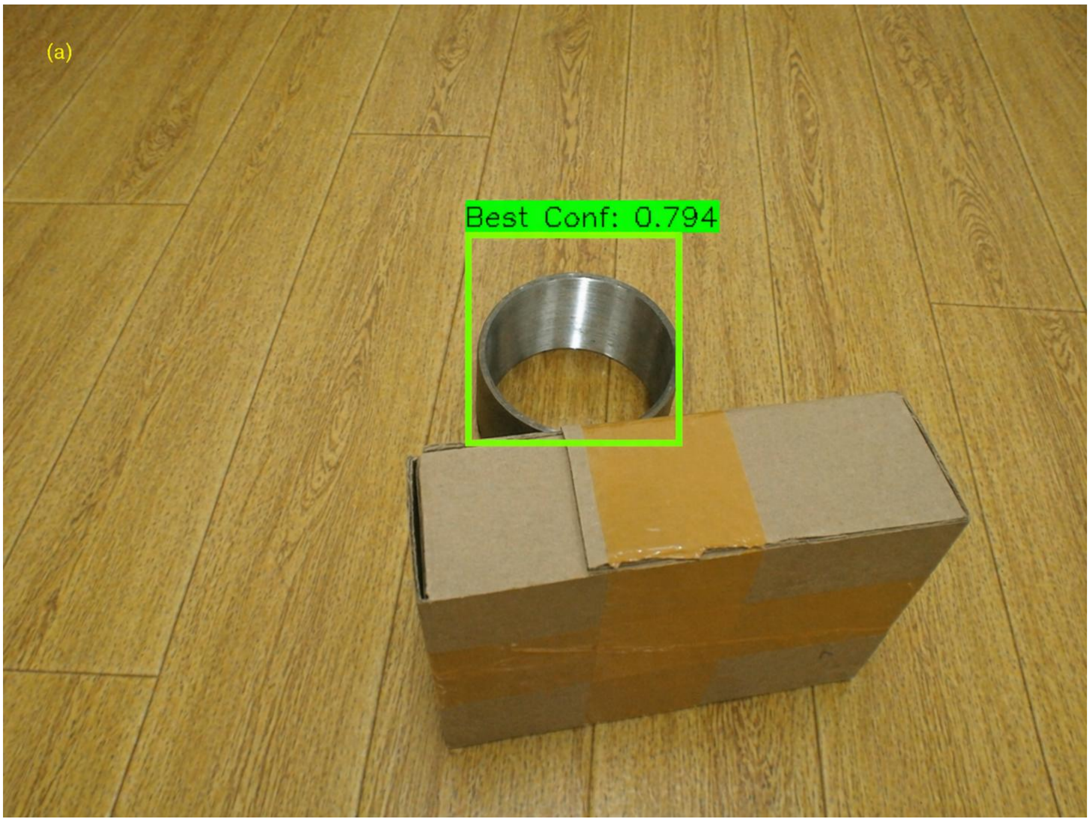

Supplementary Figure S4. Target detection result under real stereo observation.

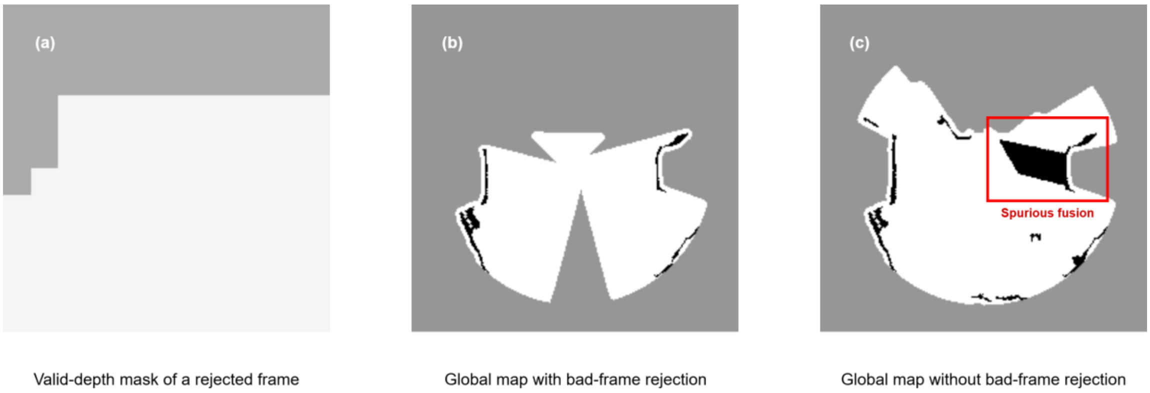

Supplementary Figure S5. Effect of quality-gated observation screening on real-scene map fusion.

Supplementary Table S10. Representative first-NBV candidate ranking without semantic guidance

Rank	Candidate	Exploration Gain	Clearance Bonus	Semantic Bonus	Total Score	Status
1	left_front	0.556	1.000	0.000	2.746	Selected
2	left_40	0.424	0.965	0.000	2.667	Rejected
3	left_back	0.418	1.000	0.000	2.644	Rejected
—	right_front	0.260	0.978	0.000	2.319	Rejected

Note: This table presents one geometry-only first-NBV example. The semantic bonus is zero, and ranking is determined by geometric exploration gain and safety-related terms.

Supplementary Table S11. Representative first-NBV candidate ranking with semantic triggering

Rank	Candidate	Exploration Gain	Clearance Bonus	Semantic Bonus	Total Score	Status
1	right_40	0.435	0.964	+0.729	3.398	Selected
2	left_front	0.556	1.000	+0.601	3.347	Rejected
3	right_front	0.447	0.950	+0.601	3.161	Rejected
—	left_40	0.424	0.966	-0.729	1.941	Rejected

Note: This table presents one semantic-guided first-NBV example. The semantic bonus re-ranks feasible candidates but does not replace geometric exploration.

Supplementary Table S12. Rejected real-scene views and rejection reasons

Frame ID	Rejection reason	Related metric	Included in global fusion
view_01	obstacle_dominant_view	roi_valid_ratio, obs_ratio_all	No
view_03	central_roi_visibility_too_low	roi_valid_ratio	No
view_05	obstacle_dominant_view	roi_valid_ratio, obs_ratio_all	No

Note: This table lists the rejected views in Real-Scene-2 together with their rejection reasons and the corresponding quality indicators. Rejected frames were not used for subsequent global map fusion. The results show that rejected views are mainly associated with low central-region visibility or obstacle-dominant observations.

Supplementary Table S13. Quality-gated observation screening results in real scenes

Scene	Total Views	Valid Fused Views (/6)	Initial Global Map Built	First NBV Generated
Real Scene 1	6	6	Yes	Yes
Real Scene 2	6	3	Yes	Yes

Note: Even when some views are rejected, the system completes initial mapping and first-NBV generation using the remaining valid observations.
